# Cucumber or grapes?—Context effects in multimedia learning

**DOI:** 10.3389/fpsyg.2025.1480935

**Published:** 2025-03-19

**Authors:** Tina Seufert, Ulrike Magner, Jurij von Randow

**Affiliations:** Department for Learning and Instruction, Ulm University, Ulm, Germany

**Keywords:** multimedia principle, situational interest, cognitive load, context effect, affective states

## Abstract

**Introduction:**

The multimedia principle states that learning with text and pictures is better than learning with text only. It might also depend on the context, in particular on the material the neighbour is learning with - which might look much more interesting.

**Methods:**

Therefore, in our experiments (*n* = 48; 47) we analysed whether learning outcomes, situational interest and extraneous load depend on the learning material (text versus text with pictures) and the context (the neighbour learns with either the same or different material). In the second study, we additionally analysed the influence of the learner’s affective state. In particular, we analysed with concurrent hypotheses whether affect is a consequence of material and context, whether effects of material and context on learning outcomes and interest are mediated by negative affect, or whether negative affect prior to learning is a moderator of material and context effects.

**Results:**

The multimedia effect was replicated for all dependent variables. This is particularly interesting because in classical multimedia design studies the effect has only been shown for learning outcomes. The fact that the addition of pictures also has a positive effect on situational interest and on the experienced extraneous load is of additional interest. The interaction between material and context was significant for extraneous load (Exp.1) and for situational interest (Exp.2). The role of affect could not be clarified.

**Discussion:**

Overall, it seems relevant to consider not only the learning material, but also the context in which the material is provided. Even if the effects of context are not consistent for all learning parameters, situational interest was strongly influenced by context when inequality was particularly salient.

## 1 Introduction

Imagine a situation at school where the teacher hands out different sheets of paper with assignments. The sheets look very different and not everyone gets the same: one might contain text and maybe tables or formulas, the other might contain text and additional colored pictures. How would the students react to these different materials? Perhaps the one with the less attractive material would be disappointed or frustrated and also less interested, while the one with the pictures would be particularly interested. The different appraisals arise from the comparison in this contrasting situation. A prominent study of this phenomenon, which we have used as an inspiration, although it is not entirely comparable, was carried out by Brosnan and de Waal (2003) with Capuchin monkeys. The monkeys were rewarded for their effort in a simple task with either cucumbers or grapes, the latter being preferred by the monkeys. Their valuation of the cucumber as a reward was still positive as long as they did not observe neighboring monkeys being rewarded with the more valued grapes. In this case, the unequal reward is perceived as unfair and the monkeys react with offensive negative affect and refusal to continue with the task. Thus, not only were emotional and motivational aspects affected, but also the monkeys’ performance.

Inspired by this work, the present study aims to identify such contextual effects in a learning situation. We analyze whether the design of learning material, in particular the use of pictures in addition to text, can also be used as a “grape” in contrast to a “cucumber,” i.e., a text without such additional pictures. We know from multimedia learning research that adding pictures to text supports learning processes, the so-called multimedia principle (Mayer, 2021a) which also holds true for problem solving (Hu et al., 2021). However, we want to analyze whether a contrasting context would accentuate this effect: while learners with the preferred learning material would learn even better when they perceive the others’ “cucumber,” learners with less supportive text-only material might actually perform worse when confronted with the neighbors’ “grape.” We are aware that providing a reward which is more or less valuable like in the study with monkeys is not the same as providing learners with different preferably learning materials. This is why we use the monkey experiment as an initial food for thought and not as a fully transferable analogy.

Situations in which learners receive different learning materials may not yet play a special role in traditional teaching situations, but modern learning settings in which adaptive technologies are used, for example, could certainly lead to learners receiving different learning materials depending on their different needs or starting situation. This possibility is currently becoming particularly relevant due to the significantly increased use of artificial intelligence in education. The new technology makes it very easy for teachers to adapt learning materials individually to learners and, for example, to illustrate them, simplify texts and so on. With this study, we want to investigate how these potentially different learning materials, and thus the context, affect learning.

To explain the effects of the context on learning, we need to take into account the cognitive processes involved in learning from text and pictures, the characteristics of the contrasting contexts, and the learners’ motivational and affective states.

## 2 How the design of learning materials affects learning

Learning often involves the challenge of understanding objects or concepts. Based on the externally provided information, learners have to build a mental representation of these concepts. Common models of learning from text and images describe this process as the construction of a mental model that is analogous to the concept to be learned (Mayer, 2021b; Schnotz and Bannert, 2003). Schnotz and Bannert's (2003) model of integrative text and picture comprehension (ITPC) explains how pictorial representations support the construction of mental models. Pictures are external representations that are also analog and thus facilitate the construction of an internal analog representation. They form a kind of mental frame in which learners can fill in additional details, for example from an accompanying text or from their existing knowledge (Schüler et al., 2015). The mental model thus contains integrated information from text and image. By processing verbal and pictorial information together, the learner uses the verbal and pictorial memory systems, i.e., the information is dual encoded. Based on Paivio’s dual coding theory (Paivio, 1986), this dual coding of information increases the likelihood of retrieving information from long-term memory. However, the positive effect on conceptual understanding or even knowledge transfer depends on the aforementioned integration of textual and pictorial information into a coherent mental model (Mayer, 2009; see also Scheiter et al., 2017; Seufert, 2003).

Many studies have confirmed the so-called multimedia principle, with better learning outcomes for learners who learn with text accompanied by pictures than for learners who learn with text alone (Mayer, 2021a). Another indicator that images can facilitate cognitive processing is the perceived cognitive load of learners. Based on cognitive load theory (Sweller et al., 1998), extraneous cognitive load results from an inappropriate instructional format. For example, having to construct an analog mental representation based only on text, i.e., without the direct scaffolding of an analog picture, can be seen as a source of extraneous load (Mayer and Moreno, 2003).

Apart from the positive effects on learning outcomes and cognitive load, it could also be assumed that text with pictures may affect learner motivation by making learning materials look more enjoyable and interesting. Thus, it seems plausible that pictures accompanying text could increase learners’ situational interest. Indeed, studies by Lenzner et al. (2013) and Magner et al. (2014) showed that learners rated multimedia learning materials as more interesting than materials with text alone. This is particularly important as interest is a crucial aspect of motivation (Deci, 1992) that can facilitate cognitive processing, as suggested by Moreno’s cognitive affective theory of multimedia learning (CATML; Moreno, 2006). In particular, learners’ situational interest, i.e., the learner’s current state of interest based on current situational aspects (Hidi and Anderson, 1992), positively influences learning processes. This can be explained by Mitchell’s differentiation of situational interest into two components, the catch and the hold component (Mitchell, 1993). While the catch component may attract learners’ attention and curiosity due to its salience, the hold component promotes sustained attentional focus, which in turn enables deeper learning processes. Harackiewicz et al. (2008) emphasize that only those aspects that are relevant to the learner can serve as a hold component. Thus, pictures accompanying a text could be a trigger of situational interest in the sense of a catch component. However, the image has to be relevant to the learning content and cannot simply be decorative in order to maintain the learner’s interest. The idea of a “faster” and more emotionally driven catch component in addition to a more cognitive hold component is also reflected in Schiefele’s differentiation of situational interest into an emotional and a cognitive component. The learner may find the object or topic ‘funny’ or ‘fascinating’, which can be seen as the emotional component. The cognitive component adds the non-emotional evaluation of the object or topic as ‘useful’ or ‘structured’. As both aspects are constituents of situational interest, they should both be addressed when measuring learners’ state of interest (Schiefele, 2009).

Learners evaluate objects or topics in their current situation and thus situational interest can arise. This interplay between the learner and the situation is also reflected in the definition of interest as an interaction between a person and an object in the environment (e.g., Boekaerts and Boscolo, 2002). Thus, we also need to consider the context of learning. If a student is working with material that he or she finds neutral or even positive, this opinion might change if a student next to him or her is working with material that seems much more or much less interesting. Thus, a context effect occurs, i.e., situational interest in one’s own learning material may be reduced or increased in this context, and this may also affect learning outcomes (Bless et al., 2004).

## 3 Effects of the context on learning

Context effect is defined as the perception of an object that is influenced by the current circumstances (Bless et al., 2004).

In educational settings, there are well-known context effects, such as the big-fish-small-pond effect (Marsh, 1987). Pupils evaluate their own abilities in relation to the abilities of their peers. They feel more capable when they perform better than their peers, or their self-concept suffers when they are less capable than their peers.

All these studies report effects on the perception of a situation, leading to adjusted evaluations or self-concepts. So it seems plausible that learners’ interest ratings for an object might be affected by context. But can we also assume that context effects actually affect learning outcomes? If learners rate learning materials as more interesting, this could affect their learning outcomes. There are several studies that report a positive relationship between interest and learning performance in school (for a review see Schiefele, 2009).

Another line of argument could be that contrasting contexts may impose a cognitive load on learners because they may irritate learners (Schäffter, 1997). Irritation could also arise from motivational or affective responses to the situation, especially if one feels inferior. These emotions could then also cause distraction and thus increased extraneous cognitive load, which could also affect learning outcomes (Sweller, 2010). Plass and Kalyuga (2019) also argue that emotions—which could be the consequence of a contrasting context—are task-irrelevant and hence cause extraneous cognitive load with possible indirect effects on learning performance. Emotions could also directly influence learning outcomes (Goetz et al., 2012). Overall, contrasting contexts seem to have the potential to influence learners’ evaluation of learning material, perceived cognitive load and learning outcomes.

## 4 Effects of affect in contrasting contexts

**Figure 1:**
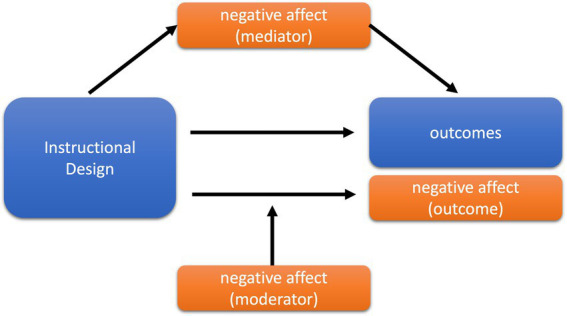
Three possible effects of negative affect in instructional design.

The learner’s affect plays a crucial role in the impact of contexts. The three possible ways in which it can be influenced can be seen in Figure 1. Each is based on a contrasting context in which the learner sees him/herself in an inferior situation. In our introductory example, this would be the case for the students learning only with the text sheets in contrast to their neighbors working with the illustrated sheets. The first way in which affect could play a role is as a consequence of this situation. The second way is that there is a mediating effect, i.e., the context causes negative affect and therefore the materials are rated as less interesting or learning is hindered. The third is a moderating effect of affect. The contrasting context might be particularly annoying for learners who already bring negative affect to the learning situation, so that their interest ratings or learning outcomes are particularly impaired when the context is also annoying. In this case, affect would increase the context effect. The three approaches differ in their perspective. The first two approaches see the treatment, i.e., the unfair context, as the cause of negative affect, which can either be seen as an independent consequence alongside interest rating and learning outcomes, or as a mediator of these. In contrast, the third approach sees the learner as the source of the negative effects of the context in the sense of an enhancer. There is previous research to support each of these mechanisms. A general discussion of the role of emotions, particularly with respect to their impact on cognitive load, can be found in Plass and Kalyuga (2019).

That the design of learning materials can induce affect as a consequence has been largely confirmed in studies of emotional design (Mutlu-Bayraktar, 2024; Park et al., 2015; Um et al., 2012). For example, colors or anthropomorphic shapes induced positive affect and also improved learning outcomes (Plass et al., 2014), which would support the first approach.

The second approach of affect as a mediator is supported by Moreno’s cognitive-affective theory of multimedia learning (CATML; Moreno, 2006) and her assumption of affective mediation. However, empirical evidence for such mediation is sparse (Leutner, 2014), and in particular the mediating pathway from affect to learning outcomes has yielded mixed effects, albeit mostly positive ones (Brom et al., 2017).

Whether learners’ affective state can moderate the effects of instructional design has also been investigated. For example, the study by Chung et al. (2015) showed that learners’ arousal moderated the effects of written versus spoken text in addition to an animation. According to the modality principle, written text is the less effective design variant (Low and Sweller, 2005), and only in this case did learners with high arousal levels, either negative or positive affect, outperform the calm groups. In another study by Kühl and Münzer (2019), learners who were afraid of spiders were unable to benefit from the multimedia principle, i.e., when text was accompanied by pictures of spiders.

Overall, there is evidence for all three possible mechanisms by which learners’ affect could influence the effects of different multimedia designs. Therefore, we analyze the role of learner affect as an exploratory question with three concurrent hypotheses.

## 5 Present studies

In two experiments we analyzed whether the multimedia principle depends on the context. More precisely we investigated the effects of learning material with either text only or text with pictures (multimedia principle) provided in contexts where neighbored learners either learned with the same or with different, i.e., either more or less interesting learning material (context effect). The dependent variables we analyzed were situational interest, learning outcomes and extraneous cognitive load. In the second experiment we additionally investigated the role of learners’ affect.

First, in both experiments we expected to replicate the multimedia effect (Mayer, 2021a), i.e., that learners show better learning outcomes with text and pictures than with text alone. We extend the replication with possible effects of multimedia presentation on cognitive load. We hypothesize that there is less extraneous load for illustrated text because learners do not have to construct a visual mental model without the external image as a scaffold. As we are particularly interested in motivational effects, we assumed that the multimedia presentation should lead to increased situational interest compared to text alone.

The second main assumption is to find a context effect. The multimedia effect on all three dependent variables should be particularly strong in the context condition where the learners learn with different learning materials than their neighbors: while the advantages might increase for those learners who receive the “better” learning material, the disadvantages should increase for those with the “worse” material.

The argument of all these assumptions is based on the fact that the learning material we used in our experiments is actually more interesting when pictures are provided instead of text alone. To ensure this prerequisite, we conducted a pre-study.

## 6 Pre-study

In the pre-study we analyzed whether the learning material with or without pictures actually produced different ratings of interest. This would be the precondition for the context effect. It is only when the illustrated text is perceived as being “better” that social cognitive processes are triggered. Thus, the aim was not to pre-test the scenarios we used in the main experiments, but to make sure that we were actually using “cucumbers and grapes” in terms of the interest of the material.

This is a short text to acknowledge the contributions of specific colleagues, institutions, or agencies that aided the efforts of the authors.

### 6.1 Method

#### 6.1.1 Participants and design

In the preliminary study, 23 students of a psychology seminar (5 male, 18 female; age: *M* = 21.7 years, SD = 1.4) participated in a within-subject design. The material consisted of text and no additional illustrative picture in the first round. In the second round, one picture was added to each page. Thus, the independent variable was learning material with or without pictures. For each of the six pages (three pages without pictures and three pages with pictures) the learners had to rate their situational interest as the dependent variable.

#### 6.1.2 Materials and procedure

##### 6.1.2.1 Pretests

In a short questionnaire, students were asked for their age and gender.

##### 6.1.2.2 Learning material

The material consisted of three pages with short text (265 words overall) describing a fictitious chocolate factory. On the first page there were some basic facts about the owner of the factory, its position in the region and its specialties. The illustrated version contained a map of the region where the factory can be seen on. The second page described the structure of the factory with its northern, eastern, southern and western sector and their railway connections. For each sector information was given about the color of the workers clothes, what they produce out of which raw products and what they cost (e.g., almonds from Tambuktu for 9.35 EUR for making marzipan). In the illustrated version a diagram of the sectors and connections, showing also the colors of the clothes and the products, enriched this page. The third page described how the Christmas specialty, the St. Nicolas praline is made. The picture illustrated how this praline looks like. The illustrated pages did not include additional information compared to the non-illustrated version, i.e., both versions have been informationally equivalent. The picture nevertheless made it much easier to see the structure of the company and the relations between the sectors, workers and products. Each learner received the three pages with text only first and afterwards the same learning material again with the illustrations. In the preliminary study, the focus was on the interest ratings of the material. Participants were told that they did not have to learn the content of the pages.

##### 6.1.2.3 Posttests

In the pre-study the dependent variable was learners’ situational interest, measured for each of the six pages. On each page learners were asked to rate whether they find the design of the learning material enjoyable, useful and interesting, each on a 7-point Likert scale from 1 = do absolutely not agree to 7 = do absolutely agree. The three items reflect the affective component (enjoyable) and the value component (useful) of interest as well as interest in general (interesting), based on Schiefele (1990). Thus, we obtained six measures of situational interest and its sub-components of every learner, three for the text-only and three for the illustrated version. The whole procedure with the pre-test and the rating of the six pages took about 15 min time. Participation was voluntary and took place at the beginning of a seminar in educational psychology.

### 6.2 Results

The t-test with dependent groups showed that learners actually rated the version with pictures more enjoyable, useful and interesting than the versions with text only. Descriptive data as well as the test statistics can be seen in Table 1. We use a significance level of *p* = 0.05 for all the statistical analyses in the paper.

**Table 1:** Mean (standard deviation) interest ratings for both groups with test statistics.

Variables	Text only	Text with picture	*t(22)*	*d*
Situational interest (overall)	3.37 (1.08)	4.40 (1.14)	4.49 ***	0.93
Affective component (*enjoyable*)	3.67 (1.18)	4.20 (1.37)	3.70 ***	0.42
Value component (*useful*)	2.93 (1.04)	5.00 (1.04)	8.88 ***	1.75
General (*interesting*)	3.52 (1.03)	4.01 (1.03)	3.63 ***	0.41

***p < 0.001.

Overall, participants showed greater situational interest for the illustrated version in contrast to the text-only version *t*(22) = 7.37, *p* < 0.01, *d* = 0.96. Based on these results the prerequisite for analyzing the context effect was given and the materials could be used for the two main experiments. As the effects were highly significant for all aspects of situational interest, we proceeded with the aggregated measure of situational interest in the two main experiments.

## 7 Experiment 1

In this experiment we addressed two research questions. The first one is, whether the multimedia effect (Mayer, 2021a) can be shown for learning outcomes and in addition also for situational interest and extraneous load. The second research question was whether the multimedia effect interacts with the context, i.e., whether the neighbored learner receives the same or different learning material.

As mentioned above we assumed to replicate the multimedia effect and thus to find a main effect of the learning material with better learning outcomes (H1a), higher situational interest (H2a) and less extraneous load (H3a) in the group with text and picture compared to text alone.

We also expected to find an interaction effect between the learning material (text only versus text with picture) and the context condition (neighbor with the same versus with differing learning material) for learning outcomes (H1b), situational interest (H2b) and extraneous load (H3b). For all the dependent variables we expected to find a stronger multimedia effect in the conditions where neighbored learners received different learning materials than in the conditions where neighbored learners received the same learning material.

We did not assume a main effect of context. It is not the mere fact that a neighbor has the same or different learning material that should have an effect, but the nature of this same or different material, i.e., whether it is more appropriate and interesting. This interplay would be reflected in the interaction effect to be analyzed.

### 7.1 Method

#### 7.1.1 Participants and design

**Figure 2:**
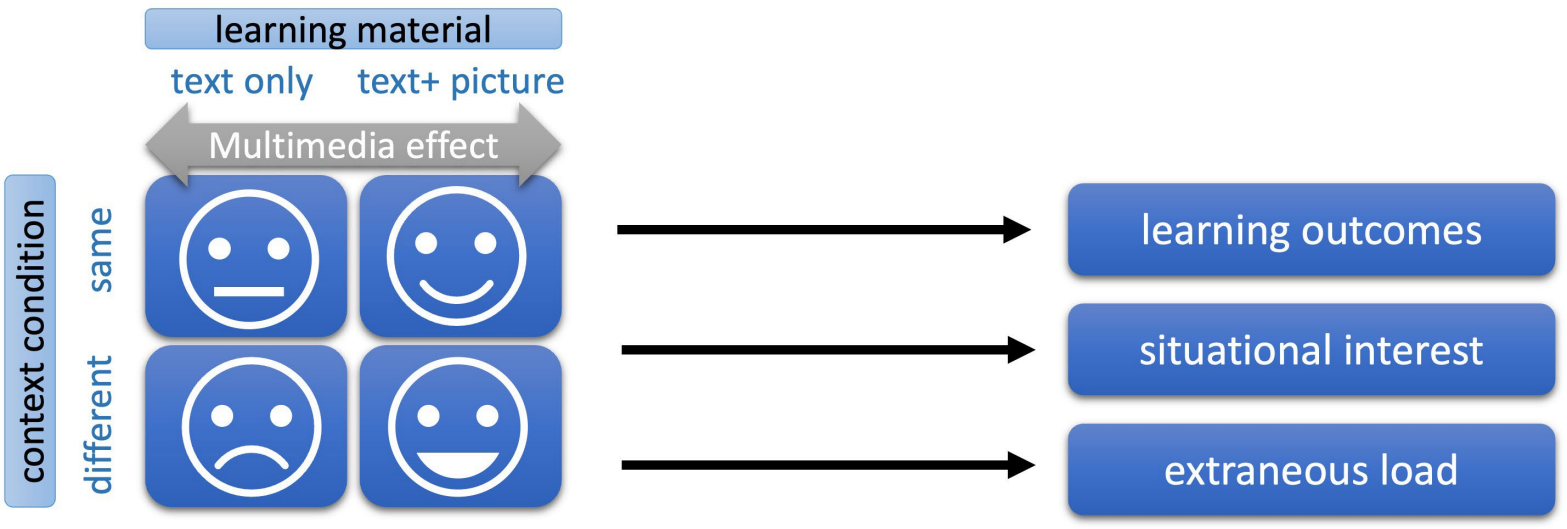
Research design of study 1.

Participants in the first experiment were 48 students of a German university with a mean age of *M* = 23.3 years (SD = 3.4), 30 of them were female. They were recruited via postings, participated voluntarily and provided an informed consent. Based on the large effect sizes of 1.39 for the multimedia effect on learning outcomes (Mayer, 2014) and the large effect size of 0.93 for situational interest in the pre-study and the assumption that context would even enhance these effects we calculated a G*Power analysis with a conservative estimated medium effect size of 0.5, which generated a sample size of 39. Participants were randomly assigned to the four experimental groups, determined by the 2 × 2 design with the factors learning material (text only versus text with picture) and context condition (neighbor with the same versus with differing learning material). The dependent measures were learning outcomes, learners’ situational interest and their perceived extraneous load. The design is depicted in Figure 2.

#### 7.1.2 Materials and procedure

Participants were informed about the procedure of the study and signed an informed consent. All participants were aware that they could withdraw their data at any point in the study without having any disadvantage. The study comprised three parts, the pretest phase, the learning phase and the post-test phase. During the pretest phase the participants had to fill out a short pretest to assess demographic data for about 3 min, followed by the learning phase including the situational interest rating which lasted about 12 min and a subsequent posttest to assess learning outcomes and the perceived extraneous load, also including a manipulation check (15 min). The session lasted 30 min overall.

##### 7.1.2.1 Pretest

In a short questionnaire at the beginning of the experiment we asked for learners’ age and gender. As we used a fictitious text, it was not necessary to assess learners’ prior knowledge. We nevertheless asked whether they already participated in previous experiments of our lab in which the same learning material had been provided. It should also be noted that the task was not high stakes for the students due to the fictional learning material.

##### 7.1.2.2 Learning material and interest rating

The learning material was the same as in the pre-study and described a fictitious chocolate firm with three chapters, each one about half a page long and either with or without an illustrating picture (either of the town, the firm’s structure, or a newly invented praline). Also, the title page of the material showed either a picture or not, so that with each glance to the neighbor the participants could see the nature of his or her material.

Situational interest was rated on the same scale from Schiefele (1990) as in the pre-study. On each of the three pages of the learning material learners had to rate their enjoyment (feeling-related aspect), the usefulness of the material (value-related aspect of interest), and their interest (overall judgment). The three components of all three points of measurements have been combined to the overall rating of situational interest with a high internal consistency over the three measurement points (aggregated *α* = 0.77; after Rodriguez and Maeda, 2006).

##### 7.1.2.3 Posttest

To ensure that learners had recognized their neighbor’s material, the first question of the posttest was whether the learning material of their neighbor had contained colored pictures. This question could be answered either yes or no and was checked and coded afterwards from the experimenter as correct or false. All participants gave the correct answer and thus no one was excluded from further analyses.

Learning outcomes have been assessed with 11 questions. Seven items measured recall of details, like: “How much does a kilo of almonds costs?.” Four items required learners apply their knowledge (e.g., “The owner of the firm wants to install cloth racks for the staff. He planned seven racks. How should he distribute these racks with regard to the staff members in each sector?”). These application tasks should be easier to answer with the illustrated learning material, as it allows the construction of a mental model from which the answers can be derived. For each question, some of them had subtasks, learners could gain 1 point for the correct answer. Overall, 23 points could be reached; results are reported in percent. The overall test reached a sufficient reliability score of *α* = 0.79.

In the last part of the posttest, we assessed learners perceived extraneous load with the three-item subscale for extraneous load of the differentiated questionnaire of Klepsch et al. (2017) with a high reliability of *α* = 0.83. An example of an item is ‘During this task, it was exhausting to find the important information’.

### 7.2 Results

**Figure 3:**
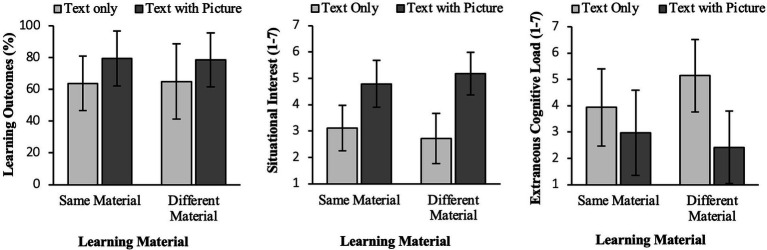
Means of the four experimental conditions of Experiment 1 for learning outcomes (in %) on the left, situational interest (range from 1-7) in the middle and extraneous cognitive load (range from 1-7) on the right.

To test our hypotheses, we conducted ANOVAs for each dependent variable with the factors learning material and context. Means and standard deviations of all analyses can be found in Table 2. Results are also shown in Figure 3.

**Table 2:** Means (standard deviations) for the dependent variables in the four experimental groups, where learners have text only or text with picture as their own learning material and neighbors learned with either the same or different learning material.

	Text only			Text with picture		
	Same material (*n* = 12)	Different material (*n* = 12)	Overall (*n* = 24)	Same material (*n* = 12)	Different material (*n* = 12)	Overall (*n* = 24)
Variables	*M (SD)*	*M (SD)*		*M (SD)*	*M (SD)*	
Learning outcomes (%)	63.77 (17.03)	64.86 (23.70)	64.31 (20.19)	79.35 (17.31)	78.62 (16.94)	78.99 (16.75)
Situational interest (1–7)	3.12 (0.86)	2.72 (0.95)	2.92 (0.91)	4.79 (0.89)	5.18 (0.80)	4.98 (0.85)
Extraneous cognitive load (1–7)	3.94 (1.47)	5.14 (1.37)	4.54 (1.52)	2.97 (1.62)	2.42 (1.38)	2.69 (1.50)

For learning outcomes we found a main effect for learning material (H1a) with higher scores for the groups with text and pictures compared to the text-only group and hence could replicate the multimedia effect, *F*(1, 44) = 7.12, *p* = 0.01, η^2^ = 0.14. We could not find an interaction effect (H1b), *F* < 1, n.s.

Concerning learners’ situational interest, we also found a strong main effect for learning material (H2a), *F*(1,44) = 66.40, *p* < 0.01, η^2^ = 0.60, indicating higher interest scores for multimedia learning material, i.e., a replication of our pre-study. The interaction was not significant (H2b), *F*(1,44) = 2.42, *p* = 0.13, η^2^ = 0.05. However, on a descriptive level one can see that the difference between the two material conditions was stronger in the context condition with different learning materials than in the context condition with the same learning materials.

For learners’ perceived extraneous load, we again found a strong main effect for the learning material (H3a), *F*(1,44) = 19.18, *p* < 0.001, η^2^ = 0.30. As expected, we found higher scores for the text-only condition. This time, we also found the expected interaction effect (H3b), *F*(1,44) = 4.29, *p* < 0.05, η^2^ = 0.09, which shows that the difference in ratings for text-only and text with picture was even stronger in the context condition with different learning material (same: MD = 0.97, SE = 0.60, *p* = 0.11; different: MD = 2.72, SE = 0.60, *p* < 0.001).

### 7.3 Discussion of study 1

The results of this experiment show that the multimedia effect could be replicated not only in terms of learning outcomes, but also in terms of learners’ situational interest and their perceived extraneous load. Thus, the picture seems to support learners on a cognitive level, as it eases information processing from extraneous affordances, but also on a motivational level by increasing interest. Consequently, learners also achieve better learning outcomes.

Regarding the second research question and the expected context effect, we had mixed results. The interaction between learning material and context was only significant for extraneous load ratings. In the groups where the learners had different learning material from their neighbors, the multimedia effect with an increase of extraneous load for the text-inly group was stronger than in the group where the neighbors had the same material. The contrast analyses also show a stronger multimedia effect for situational interest depending on the context, but for learning outcomes we could not find any context effects. As the learning outcomes were quite high overall, this could be due to a ceiling effect. However, we were still able to influence these high scores through the learning material and had a significant effect by either providing pictures or not. This leads us to conclude that the impact of context was not strong enough.

Therefore, in the next experiment we decided to increase the contextual sting by directing learners’ attention to the neighbor learning material at the beginning of the learning phase, rather than afterwards as in this study. This should make (in)equality more salient and influential throughout the learning process. We also added more difficult questions to the learning outcomes test to avoid ceiling effects. Moreover, it would be interesting to understand why the context effect occurred for extraneous load and which role learners affect could play. Hence, we investigated the aforementioned role of learners’ affect as a consequence, mediator or moderator of possible context effects.

## 8 Experiment 2

The second experiment is a replication of the first one with a more intensive implementation of the context factor. By asking the participants at the beginning of the learning phase whether the learning material of their neighbors looks different or the same as their own, we aimed to enhance the salience of equality or—more importantly—of inequality. The effect of the interaction between learning material and context was also analyzed regarding learning outcomes, situational interest and extraneous cognitive load.

The first two research questions were thus the same: we first wanted to investigate the multimedia effect for learning outcomes (H1a), situational interest (H2a) and extraneous cognitive load (H3a) and second to find out whether the multimedia effect depends on the context of learning with the same or different learning material than the neighbor (H1-3b).

In a third research question we wanted to explore the role of learners’ negative affect. As outlined above, we analyzed the role of learners’ negative affect from different perspectives, resulting in three different concurrent hypotheses (H4-6).

First, it would be possible that *negative affect is the consequence* (H4) of the treatment. More precisely, we assumed that learning with less supportive learning material, i.e., with text only can lead to more negative affect compared to supportive learning comprising text and pictures (main effect of learning material on negative affect). This enhancing or decreasing effect on negative affect might even be stronger when learners are facing inequality by observing their neighbors with the less or more supportive material (interaction effect of learning material and context on negative affect). In this case, the mere fact that the neighbor has different or the same material, irrespective of the supportive nature of the learning material could have an impact on learners’ negative effect (main effect of context on negative affect).

The second assumption was based on the first one and hypothesized that negative affect arises from the learning material per se or in interaction with the context and *mediates the effects* on learning outcomes, situational interest or extraneous cognitive load (H5). With this mediation one could explain the results found in experiment one.

The third assumption was that learners who start to learn in a negative affective state will be more strongly influenced by the material or the interplay of material and context. In this case learners *negative affect before learning would moderate the effects* on learning outcomes, situational interest or extraneous cognitive load (H6).

### 8.1 Method

#### 8.1.1 Participants and design

In our experiment 47 participants were analyzed with a mean age of *M* = 24.7 years (SD = 4.2), 34 of them were female. They were recruited via postings at a German University, participated voluntarily and provided an informed consent. We used a 2 × 2 design with the factors learning material (text only versus text with picture) and context condition (neighbor with the same versus with differing learning material). The dependent measures were again learners’ learning outcomes, their situational interest and their extraneous cognitive load. In addition to the first experiment, we assessed learners’ state of negative affect before and after the learning phase and analyzed its role as a potential outcome, mediator or moderator.

#### 8.1.2 Materials and procedure

The experiment comprised again three phases: the pretest assessed demographic data and learners negative affect at the beginning (8 min), the learning session including the manipulation check at the beginning and the situational interest rating on each page of the learning material (12 min), and the posttest session to assess learning outcomes, the perceived extraneous load as well as the negative affect after learning (20 min). The session lasted 40 min overall.

##### 8.1.2.1 Pretest

We used the same short questionnaire at the beginning of the experiment to assess learners’ age and gender. As we used the same fictitious text on a chocolate firm, it was again not necessary to assess learners’ prior knowledge but we asked for participation in previous experiments of our lab with the same learning material.

In addition to the first experiment, we assessed learners’ negative affect before and after the learning phase with the subscale for negative affect of the PANAS (positive and negative affect scale; Watson et al., 1988, translated into German by Krohne et al., 1996). The questionnaire consists of 10 items, each with one affective adjective like nervous, upset, or afraid. Learners had to rate whether they currently feel like that on a Likert scale from 1 = almost not to 5 = very much. The internal consistency of the scale was satisfying with Cronbach’s *α* = 0.87.

##### 8.1.2.2 Learning material and interest rating

We used again the same learning material describing a fictitious chocolate firm (title page plus 3 pages with text and pictures in the picture-groups) with three chapters, each one about half a page long and either with or without an illustrating picture (either of the town, the firm’s structure or a newly invented praline). Also, the title page of the material showed either a picture or not, so that with each glance to the neighbor the participants could see the nature of his or her material. In contrast to the first experiment, we strengthened the manipulation of the context factor by asking learners before they started to learn whether their neighbors learn with the same or differing materials. Even on this page with the manipulation check item we added a picture in the picture groups and had the blank page without picture in the no picture groups. Learners could also answer that they do not know whether their neighbor has the same or different material for cases where learners might not have been able to see the neighbor’s material. This option turned out to be not necessary, as all participants could rate their neighbor’s learning material and did so correctly.

Situational interest was rated with the same scale as in experiment 1 (Schiefele, 1990), again on each page with the items for enjoyment, usefulness and interest, each on a 7-point Likert scale from 1 = agree not at all to 7 = agree completely. The overall scale of situational interest comprising all three aspects for all three points of measurement was highly consistent (aggregated *α* = 0.77).

##### 8.1.2.3 Posttest

The test for learning outcomes was extended with two more items for remembering details like the name of the owner of the firm and one additional item with a transfer task on how best to produce a new chocolate, given the structure and processes of the chocolate company. Altogether it consisted of 14 tasks, 7 of them for recall of details and 7 of them for applying the concepts. The internal consistency was again satisfying with *α* = 0.76.

After the test for learning outcomes learners had again to rate their negative affect with the negative affect scale of the PANAS (Watson et al., 1988), i.e., they indicated whether they feel, e.g., upset or afraid on a 5-point Likert scale from 1 = not at all to 5 = very much. The last part of the posttest comprised again the subscale for extraneous cognitive load from the differentiated load questionnaire of Klepsch et al. (2017), which comprised three items and had a good internal consistency of *α* = 0.72.

### 8.2 Results

**Figure 4:**
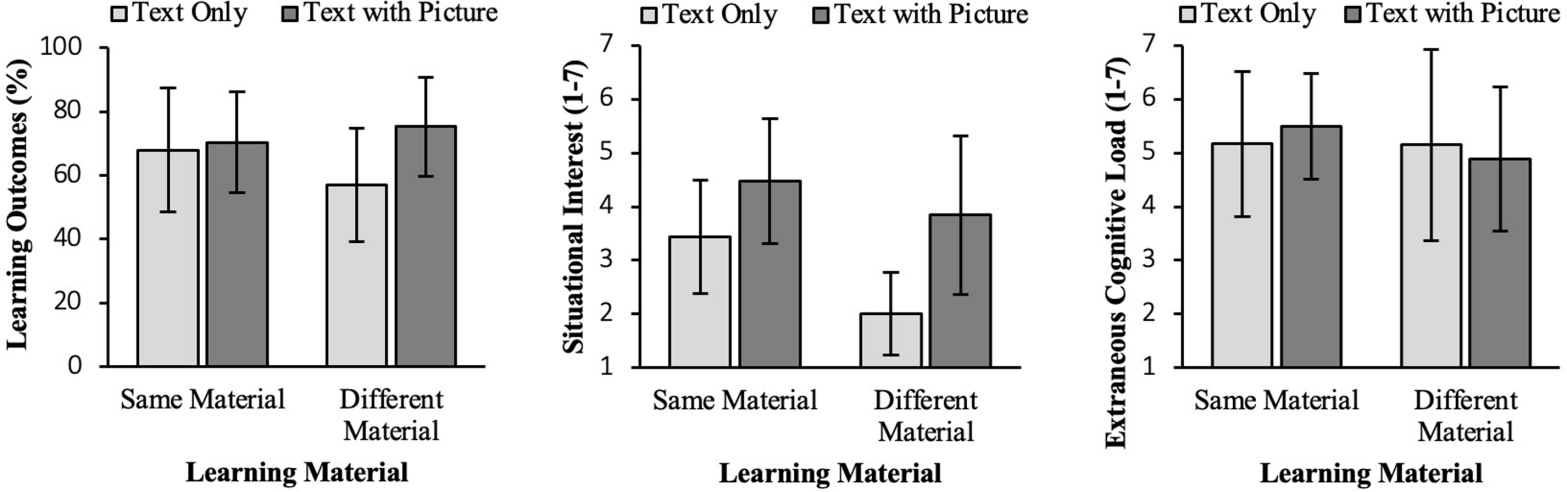
Means of the four experimental conditions of Experiment 2 for learning outcomes (in %) on the left, situational interest (range from 1-7) in the middle and extraneous cognitive load (range from 1-7) on the right.

To test our hypotheses, we conducted ANOVAs for each dependent variable with the factors learning material and context. Means and standard deviations for learning outcomes, situational interest and extraneous load can be found in Table 3. The results are also shown in Figure 4.

**Table 3:** Means (standard deviations) for the dependent variables in the four experimental groups where learners have text only or text with picture as their own learning material and neighbors learned with either the same or different learning material.

	Text only			Text with picture		
	Same material (*n* = 12)	Different material (*n* = 10)	Overall (*n* = 22)	Same material (*n* = 13)	Different material (*n* = 12)	Overall (*n* = 25)
Variables	*M (SD)*	*M (SD)*		*M (SD)*	*M (SD)*	
Learning outcomes *(%)*	67.92 (19.43)	57.00 (17.72)	62.95 (19.06)	70.38 (15.90)	75.28 (15.60)	72.73 (15.63)
Situational interest (1–7)	3.44 (1.05)	2.01 (0.77)	2.79 (1.17)	4.47 (1.16)	3.84 (1.47)	4.76 (1.01)
Extraneous cognitive load (1–7)	5.17 (1.35)	5.15 (1.78)	3.95 (1.42)	5.50 (0.98)	4.88 (1.34)	1.88 (0.83)

For learning outcomes we found a main effect for learning material (H1a) and hence could replicate the multimedia effect, *F*(1,43) = 4.25, *p* < 0.05, η^2^ = 0.09. We could not find an interaction effect (H1b), *F*(1,43) = 2.47, *p* = 0.124, η^2^ = 0.06. However, based on the hypotheses we analyzed the contrasts between text alone versus text and picture groups for each context condition, assuming stronger differences in the context condition with different learning materials of the neighbors. In fact, the contrast analysis revealed a significant difference between the groups which learned with text and picture versus text alone in the conditions with differing materials (MD = −18.28; SE = 7.36, *p* = 0.017) while learners in the group with the same materials as their neighbors showed no significant multimedia effect (MD = −2.47, SE = 6.88, *p* = 0.721).

Concerning learners situational interest, we also found a strong main effect for learning material (H2a), *F*(1,43) = 52.84, *p* < 0.001, η^2^ = 0.55, indicating higher interest scores for multimedia learning material, i.e., again a replication of the multimedia effect. The interaction was also significant (H2b), *F*(1,43) = 13.18, *p* = 0.001, η^2^ = 0.24. The difference between the two material conditions was stronger in the context condition with different learning materials (MD = −3.07, SE = 0.41, *p* < 0.001) than in the context condition with the same learning materials (MD = −1.03, SE = 0.39, *p* = 0.01).

For learners’ perceived extraneous load we again found a strong main effect for the learning material (H3a), *F*(1,43) = 37.56, *p* < 0.001, η^2^ = 0.47. As expected, learners rated the extraneous load in the text-only condition higher than in the multimedia condition. In contrast to experiment 1, we found no interaction effect (H3b), *F* < 1, n.s.

#### 8.2.1 Role of learners’ negative affect

To assess the role of learners’ negative affect, we examined three different potential pathways of effects. (H4) negative affect as an outcome, (H5) the development of negative affect during the learning phase as a mediator, or (H6) negative affect prior to learning as a moderator.

Mediation and moderation analyses were computed with the PROCESS 4.0 macro for SPSS from Hayes (2017). The affect scores before and after learning as well as the difference score are shown in Table 4 for the different experimental groups.

**Table 4:** Means (standard deviations) for the affect scores in the four experimental groups.

	Text only		Text with picture	
	Same material	Different material	Same material	Different material
Variables	*M (SD)*	*M (SD)*	*M (SD)*	*M (SD)*
Negative affect before learning (1–5)	1.32 (0.40)	1.16 (0.25)	1.22 (0.30)	1.48 (0.48)
Negative affect after learning (1–5)	1.23 (0.27)	1.27 (0.45)	1.17 (0.20)	1.43 (0.39)
Difference score	−0.9 (0.27)	0.11 (0.28)	−0.05 (0.25)	−0.05 (0.28)

##### 8.2.1.1 Affect as an outcome variable (H4)

The learning situation with different learning materials and different context situations could influence learners’ affective state in a negative way. Therefore, we calculated an ANOVA with learning material and context as independent variables and learners’ negative affect as a dependent variable with learners’ affective negative affective state before learning as a covariate. We found no main effect of the learning material, i.e., whether the learning material included text only or additional pictures did not influence learners’ affective state, *F* < 1, n.s. We nevertheless found a significant effect for the context variable, *F*(1,42) = 2.81, *p* = 0.05, η^2^ = 0.06, with higher scores of negative affect in the group where learners had neighbors with different material than in the group with learners who learned with the same material. We found no interaction effect, *F* < 1, n.s.

##### 8.2.1.2 Affect as a mediator for learning outcomes and situational interest (H5)

As just described and against our expectations, the design factors of the learning material did not influence learners’ negative affect. Thus, negative affect cannot be considered as a mediator between the treatment and learning outcomes or situational interest.

##### 8.2.1.3 Affect as a moderator for learning outcomes (H6a)

The overall regression model with learning material and context as independent factors and learners’ negative affect before learning as a moderator for learning outcomes as dependent variable revealed no overall significant result, *F*(7,39) = 1.11, *p* = 0.379, R2adj = 0.17. Also, the moderation effect, i.e., the triple interaction of the two independent variables and learners’ negative affect was not significant, *F* < 1, n.s.

##### 8.2.1.4 Affect as a moderator for situational interest (H6b)

**Figure 5:**
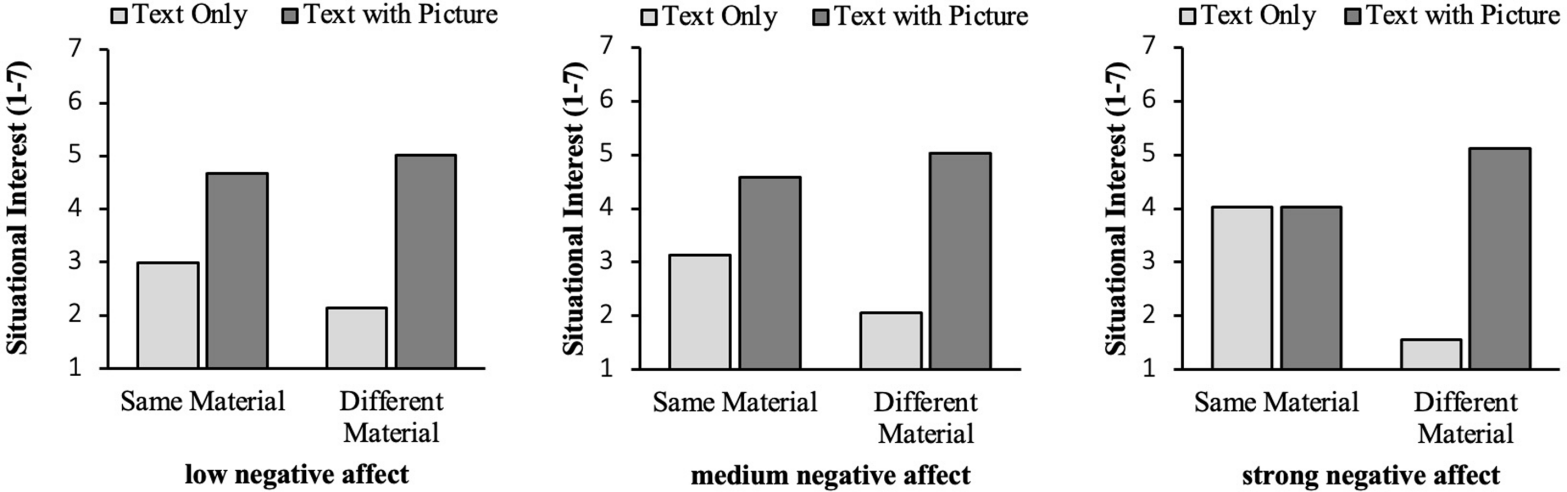
Means of the four experimental conditions for situational interest for learners with low negative affect before learning on the left (- 1 SD), medium negative affect in the middle and strong negative affect (+ 1 SD) on the right.

The moderation model for situational interest as dependent variable revealed a significant overall result, *F*(7, 39) = 10.16, *p* < 0.001, R2adj = 0.65. Learners’ negative affect nevertheless did not significantly moderate the interaction of the two design factors on situational interest, *F*(1, 39) = 3.14, *p* = 0.084. The descriptive analysis of learners with different levels of negative affect nevertheless showed an interesting pattern (see Figure 5; the groups were built *post hoc* for visualizing the effects, but the analysis was run with the continuous variable for negative affect). The difference in situational interest for learners with text and picture versus text only was always smaller when neighbors had the same material instead of different materials. This gap between same and different context learners widened continuously with increasing negative affect. In the fictitious group of learners with highly negative affect, the rating between the two material types even vanished when neighbors had the same learning material while it was most pronounced when neighbors had different materials. Thus, learners with strong negative affect were more likely than learners with a medium or low level of negative affect to find text-only material much less interesting compared to text and picture material.

### 8.3 Discussion of study 2

In the second experiment we could replicate the multimedia effect for all dependent measures: learners showed higher learning outcomes, less extraneous and more situational interest when they learned with text and picture than with text alone. This is in line with the results of the first experiment and many other studies on the multimedia effect (for an overview see Mayer, 2021a).

Regarding the influence of the context, we aimed at intensifying the perceived difference of contexts for the learners by asking them to check for their neighbors’ material right at the beginning of the learning phase. With this intensified sensitivity for the context during learning, we assumed to find stronger context effect for learning outcomes and situational interest, which we could not substantiate in the first experiment. In fact, we could demonstrate the context effect for learning outcomes with different patterns for the two groups with either the same or different learning material than their neighbors and with an overall significant interaction for situational interest. In both cases we could show that the multimedia effect was more pronounced when neighbored learners had different instead of the same learning material. However, in this second study we could not replicate the context effect for extraneous load. In this study, the rating for extraneous load which followed after the posttest for learning outcomes the retrospective rating was less influenced by the inequal context as this was made salient at the very beginning of the learning phase. Thus, the potential “stinging” effect might have faded. The nature of the learning material, i.e., whether it contained texts or texts and pictures in contrast was still present at the learner’s retrospective evaluation of the instructional quality. We hence assume that we might have found context effects for all the dependent measures if we would have asked learners to check for their neighbors’ material – and thus to set the sting – at the beginning of the learning phase and at the end. With this measure we would ensure that the context is salient at all points of measurement and would affect the overall learning phase. Moreover, with a larger sample the effects might also have been significant. Finally, it has to be noted, that the timing of the contrasting sting should be chosen carefully. It can bias and overshadow the learning process and the completion of the post-test tasks. Learning more about the potential confounding effects and their nature in terms of cognitive, metacognitive, affective or motivational effects would be an interesting follow-up study.

Whether learners’ affect play a role for different instructional material and the context was also addressed in this study in an exploratory way. We analyzed three different influencing paths of negative affect, as a consequence, a mediator or a moderator. We could not substantiate effects of the learning material and the context on learners’ negative affect. Thus, negative affect could also be discarded as a potential mediator of the effects on learning outcomes, situational interest and cognitive load. These results are quite unexpected, but one reason might be the nature of the affect measure. We asked for negative affect before and after learning but not during the process of learning and particularly not directly after inducing the context factor. It seems plausible that with more intensive measures over time one might be able to identify critical situations and thus more directly contextual effects on learners’ affect.

However, the third mechanism of affective effects, the moderation, turned out to be valid. When learners started the learning session with an already strong negative affect the impact of the context is meaningful regarding their situational interest. However, this moderating impact did not translate into negative effects on learning outcomes. Obviously, learners could distinguish that beside their impeded motivation they were still willing to invest sufficient effort to master the task at hand. This was solved quite well in general in all experimental groups – particularly in those with additional pictures.

Overall, we can conclude from the second experiment that the instructional material has the most direct impact on cognitive and motivational variables, but that the context on the one hand and learners affective state on the other hand can moderate these instructional effects. It seems thus worthwhile to either control the context or to ensure equal conditions for all learners and to foster positive emotions before learning.

## 9 General discussion and conclusions

In two studies we analyzed whether the effects of multimedia learning materials depend on the context in which they are provided. Over both studies, the results consistently approved the multimedia principle for learning outcomes and also on learner’s interest. This finding supports the idea of Moreno’s CATML (2006), that not only cognitive processes are affected but also learners’ motivation. Furthermore, we could also substantiate the multimedia principle for cognitive load: learners reported an increased level of extraneous cognitive load when they learned with text only compared to those who learned with an additional picture. To our best knowledge, this effect has actually not been proven until now for the multimedia effect. However, it is clearly in line with other effects that refer to the cognitive theory of multimedia learning (CTML; Mayer, 2009) or the integrated model of text and picture comprehension (ITPC; Schnotz and Bannert, 2003). When the crucial learning processes of selection, organization and integration – and most importantly the construction of a mental model – are supported by the learning material, learners should not only show increased learning outcomes but also a decrease in extraneous cognitive load. With regard to cognitive load theory, it would nevertheless be also interesting to not only focus on passive aspects of load, i.e., those requirements arising from external sources like the learning material or its design, in case of extraneous load (Klepsch and Seufert, 2021). As learners can deliberately decide how much effort they would like to invest, the active aspects, i.e., those that are germane to the task and arise from the learner him or herself could also play a role in this study. The first mechanism for a possible increase in germane load would be that concomitantly with the decrease in extraneous load learners would have more resources left which they could invest in terms of germane load. But still learners need to be willing to invest the freed-up resources and this is where the second mechanism would come into play. In a favorable context where I find myself with more interesting and likeable learning material than my neighbor, I might be willing to invest more of these free resources. Of course, this effect would reverse for those learners who have the less favorable learning material. Thus, the invest of germane load and effort could be an indicator for the motivational effect which arises from the context. Learners’ interest in the topic itself (not just the way it was presented, as measured and analyzed in the present study) may be an interesting moderating variable for future studies. In line with this, also a differentiation in surface and deep learning processes could be fruitful to learn more about the interplay of motivation and learning. Learners who are encouraged might invest particular effort in deep learning processes while those who are discouraged might only focus on surface level learning processes.

Another aspect that has not yet been discussed is that the effects of context, evaluation of the learning material and possible adaptations in terms of motivation and effort could also be driven by a metacognitive process triggered by the comparison of the learning material. The learner could consciously decide that it is not worth putting effort into learning material that is less or more suitable or favorable. What students actually think when they compare, and whether the ratings of effort, interest or actual performance can be explained by these thoughts, needs to be further investigated in future studies.

However, despite the impressive evidence for the multimedia effect on different effect measures and the well-grounded theoretical explanations, one can still ask what the driving mechanism for the multimedia effect is. Is it the eased processing of textual information when the picture helps to visualize, e.g., unknown, difficult or abstract terms? Or is the construction of the mental model eased because the picture is analog in format and thus learners can simply copy the external picture into an internal one? While we can assume that with the decreased extraneous load the eased processing is plausible, we cannot be sure whether learners in the text only condition really struggle in constructing a mental model themselves without the external blueprint. Maybe the mechanism is not of cognitive nature at all but rather motivationally driven? All these possible processes or mediating forces, like extraneous or germane load, situational interest, attention guidance or insights into mental model constructing, e.g., by thinking aloud, need to be investigated repeatedly to detect the crucial mechanism and thus to refine the theoretical framework for the multimedia effect.

The studies broadened the perspective on multimedia effect not only with regard to the context but also with regard to learners’ affective state. While the “sting” of the unequal context obviously was not strong enough to cause negative affect and thus to function as a mediator, we found evidence that learners initial affective state moderates the effects of the learning material and context with regard to situational interest. In line with the argument above, it thus would be interesting to learn how the affective state of learners influence the learning process. This could be figured out maybe by using qualitative measures like prompted recall (Bannert and Mengelkamp, 2008). Such a qualitative interview technique could also help to detect the individual reactions of inequity and the appraisal of this situation.

Beside learners’ affective state other learner characteristics could also be taken into account like the need for equal treatment and fairness or further cognitive variables like learners’ prior knowledge. In these studies, we have chosen a fictitious learning material to rule out possible prior knowledge effects and to analyze the mere effects of treatment and context. However, in future studies it would be interesting to analyze potential effects of prior knowledge systematically as an additional factor. It seems plausible that learners with higher expertise might be less affected by non-supportive treatments or might even suffer from additional pictures which they do not need, as is suggested by the expertise reversal effect. Also, contextual effects might be less relevant as learners can rely on what they already know and might not be too dependent on external factors as long as the necessary information is provided.

Overall, we can conclude that with these two studies we have first evidence that the context in which learning material is provided can have substantial effects—not only on learning outcomes but also on learners’ motivation and that learners affective state at the beginning of the learning phase is influential. In future studies, other learning materials and instructional design effects could be analyzed in order to find out whether they also induce the effect of only getting cucumber while others receive grapes.

## Data Availability

The raw data supporting the conclusions of this article will be made available by the authors, without undue reservation.
